# Specific knockout of notch-1 attenuates non-alcoholic fatty liver disease by promoting SHP2 phosphorylation

**DOI:** 10.18632/aging.205305

**Published:** 2023-12-13

**Authors:** Qian Gao, Yonggang Lu, Weiling Zhou

**Affiliations:** 1 Department of Endocrine and Metabolic Diseases, The Fourth Hospital of Hebei Medical University, Shijiazhuang 050000, China; 2 Clinical Laboratory, Hebei General Hospital, Shijiazhuang 050000, China

**Keywords:** NAFLD, Notch-1, SHP2, inflammation, lipid deposition

## Abstract

Objective: To investigate the effect of Notch-1 signaling on NAFLD and its molecular mechanism.

Methods: The lipid deposition in liver tissues was detected by oil red O staining. Western blotting was performed to detect the expressions of SREBP1C, SREBP2, LXR, IL-1β, IL-18, NLRP3, Notch-1, NOX2, NOX4, p-PI3K and p-SHP2 in macrophages, and the expressions of ALIX, CD9, IL-1β and SREBP1C in exosomes. Macrophages in the Notch-1^MAC-KO^ group and Notch-1^WT^ group were treated with FFA, and those in the Notch-1^WT^+FFA group and Notch-1^MAC-KO^+FFA group were treated with SHP2 inhibitors PHPS1 and Relaxin.

Results: It was observed by oil red O staining that lipid deposition in mice with NAFLD was reduced in the Notch-1^MAC-KO^ group. The results of Western blotting showed that the expressions of ALIX, CD9, IL-1β and SREBP1C in macrophage exosomes were significantly lower in the Notch-1^MAC-KO^ group than in the Notch-1^WT^ group. In macrophages, the expressions of SREBP1C, SREBP2, LXR, IL-1β, IL-18, Notch-1, NOX2, NOX4 and p-PI3K significantly decreased, while the expression of p-SHP2 significantly increased in the Notch-1^MAC-KO^ group compared with the Notch-1^WT^ group. The Notch-1^MAC-KO^+FFA group had significantly decreased expressions of SREBP1C, NLRP3, IL-1β, IL-18, SREBP2, NOX2, NOX4 and p-PI3K and a significantly increased expression of p-SHP2 compared with the Notch-1^WT^+FFA group. However, the differences in the above proteins were all eliminated after PHPS1 and Relaxin were added.

Conclusion: Specific knockout of Notch-1 attenuates NAFLD, and reduces inflammation and lipid deposition in the liver by promoting SHP2 phosphorylation.

## INTRODUCTION

NAFLD is a common chronic liver injury characterized by severe hepatocellular steatosis with inflammation and lipid deposition and no history of alcoholic liver injury. With the increasing prevalence of obesity, the morbidity of NAFLD is on the rise globally [1, 2]. Due to high-calorie and HFD, NAFLD has rapidly become a global public health problem [3]. However, no effective drugs are available for NAFLD so far [2, 4]. Therefore, it is necessary to study the molecular pathophysiology of NAFLD for developing new therapeutic strategies.

NAFLD has a complex and multifactorial pathogenesis, and severe hepatocellular steatosis with inflammation plays an important role in the development of NAFLD [5–7]. There is new evidence that lipid deposition in hepatocytes triggers inflammation and apoptosis in hepatocytes, ultimately leading to NAFLD [8, 9]. Exosomes are extracellular vesicles secreted by a variety of cells, including macrophages, ranging in size from 30 to 100 nm with lipid bilayer membranes [10, 11]. Accumulating evidence suggests that exosomes are considered mediators of cell-to-cell communication and can efficiently transfer DNA, proteins, and lipids to recipient cells, thereby playing a vital role in cellular communication [12–14]. In this study, the potential role of macrophage-derived exosomes in NAFLD and their underlying mechanisms were explored.

The Notch signaling pathway is a conserved pathway involved in cell fate determination in embryonic development, which controls cell proliferation and differentiation [15]. As a member of the Notch receptor family, Notch-1 is a key regulator of chronic inflammation and lipogenesis, which may be associated with NAFLD [16–19]. Therefore, Notch-1 signaling may be a potential therapeutic target for NAFLD.

It has been verified that Notch-1 signaling has positive feedback on ROS, which inhibits the phosphorylation of SHP2. SHP2, encoded by the human PTPN11 gene, is a ubiquitously expressed non-receptor PTP that consists of N-SH2 and C-SH2 domains. These two domains are important for subcellular localization of SHP2, while the PTP domain is critical for its enzymatic activity. SHP2 can respond to signaling of growth factors and cytokines, and plays a role in immune and inflammatory responses. For example, by inhibiting ANT3 and mitochondrial dysfunction, SHP2 can coordinate an internal regulatory circuit to limit the overactivation of NLRP3 inflammasomes. These findings have important implications for a deeper understanding of the role of SHP2 in cell function and disease development [20].

However, the effect of macrophage-derived Notch-1 signaling on NAFLD and its molecular mechanism remain unknown. Therefore, the effect of specific knockout of Notch-1 on macrophages in NAFLD mice was explored and its molecular mechanism was elucidated in this paper.

## MATERIALS AND METHODS

### Animals

Notch-1^flox/flox^ mice and mice expressing Cre recombinase under the control of the lysozyme 2 (Lyz2) promoter (Lyz2 or LysM-Cre) were purchased from Shanghai Kinbio Tech. Co., Ltd (China). Notch-1^flox/flox^ mice and LysM-Cre^+/−^ mice were crossed to generate Notch-1^MAC-KO^ mice, and Notch-1^flox/flox^ mice and LysM-Cre^−/−^ mice were crossed to generate Notch-1^WT^ mice. All mice were fed an HFD consisting of 20% fat, 0.05% cholesterol, 15% lard, 3% sugar, 1% porcine bile, 5% egg yolk powder, 20% egg yolk powder, and 0.05% vitamins for 6 weeks at room temperature and 12/12 h light-dark cycle to induce NAFLD. All experiments were performed in accordance with the Guidelines for the Care and Use of Laboratory Animals and were approved by the Fourth Hospital of Hebei Medical University (Hebei, China).

### Oil red O staining

After the mice were anesthetized with ketamine, fresh liver tissues were harvested from the right lobe, fixed in a 4% paraformaldehyde solution (Sigma-Aldrich, USA) for 12 h, dehydrated in ethanol, and embedded in paraffin. Then paraffin-embedded sections (6 μm) were stained with Oil Red O Staining Kits (Shanghai Yope Biotech Inc., China), followed by histological examination under an inverted fluorescence microscope.

### Flow sorting

Liver tissues were aseptically isolated in each group, washed with D-PBS to remove blood stains, and cut into 1 mm^3^ tissue pieces with sterile scissors. The tissue pieces were digested several times (5 min each time) with a mixture of 0.2% type IV collagenase and 0.25% trypsin at 37°C in a 50-mL conical flask using a sterile small magnetic stirring bar on a stirrer until they disappeared. After the termination of digestion with a complete medium, the supernatant was collected, filtered through a 200-mesh sieve, resuspended with cell staining buffer (1 × PBS containing 1% BSA) and placed in a refrigerator at 4°C. After the cell density was adjusted to 1 × 10^6^/100 μL, they were incubated with Rabbit Monoclonal (EPR23917-164) to CD68 (ab283654, Abcam, UK) and Goat Anti-Rabbit IgG H&L (Alexa Fluor^®^ 488) (ab150077, Abcam) for 30 min away from light. Finally, the cells were analyzed and sorted using BD FACSAria™ III. Cell debris and clumps were removed, and CD68-positive macrophages were purified using markers.

### Extraction of BMDMs

Bone marrow cells were obtained from the femur and tibia of C57BL/6 mice, cultured in DMEM (10% and 20% hFF) supplemented with 15% fetal bovine serum (Gibco, USA), 100 U/mL penicillin G, 100 μg/mL streptomycin, L-glutamine (2 mm, Sigma, USA) and 20% L929 conditioned medium, and stimulated to differentiate into BMDMs using macrophage colony-stimulating factors. BMDMs were cultured in a 24-well plate for 6 days at a concentration of 5 × 10^4^ cells/well in a humidified atmosphere containing 5% CO_2_ and 95% air at 37°C.

### Isolation of exosomes

Exosomes were isolated by using the Exosome Isolation Kit (Thermo Fisher Scientific, USA) according to the manufacturer’s instructions. First, the culture supernatant of macrophages was centrifuged at 10,000 g for 20 min at 4°C, and filtered through a membrane filter (EMD Millipore, USA). Then the sample was mixed with Total Exosome Isolation Reagent (Invitrogen, USA) and incubated at 4°C for 24 h, followed by centrifugation at 500 × g for 30 min at 4°C. Finally, the exosome pellets were washed and resuspended in PBS.

### Cell culture

BMDMs and exosomes were cultured in DMEM supplemented with 10% exosome-free fetal bovine serum, 100 U/mL penicillin, and 100 mg/mL streptomycin at 37°C and 5% CO_2_/95% air. Isolated macrophages were plated in duplicate four times in a 48-well plate (2.5 × 10^5^ cells/well, 5% CO_2_, 37°C), and then the supernatant was collected and added with fresh medium containing 10% exosome-free fetal bovine serum. Besides, macrophages were divided into Notch-1^MAC-KO^ group, Notch-1^WT^ group, Notch-1^WT^+FFA group, Notch-1^MAC-KO^+FFA group, Notch-1^WT^+FFA+PHPS1 group, Notch-1^MAC-KO^+FFA+PHPS1 group, Notch-1^WT^+FFA+Relaxin group and Notch-1^MAC-KO^+FFA+Relaxin group.

### Western blotting

The cells were lysed with ice-cold RIPA lysis buffer (Beyotime Biotechnology, China) containing phosphatase and protease inhibitors (Thermo Fisher Scientific) and centrifuged at 12,000 g for 40 min at 4°C to extract the total protein. The total protein was quantified using the BCA Protein Quantification Kit (Beyotime). Then the protein (40–80 μg per well) was separated by 10% SDS-PAGE and transferred onto PVDF membranes. The membrane was incubated with primary antibodies against SREBP1C, SREBP2, LXR, IL-1β, IL-18, p-SHP2, ALIX, CD9, NLRP3, NOX2, NOX4, p-PI3K, Notch-1, and GAPDH. After washing with TBST (TBS with 0.05% Tween-20), the membrane was incubated with the corresponding horseradish peroxidase-conjugated secondary antibodies for 2 h. Finally, protein bands were visualized using enhanced chemiluminescence and analyzed by ImageJ.

### Statistical analysis

All the results were expressed as mean ± standard deviation and analyzed by SPSS 22.0. Differences were compared by one-way ANOVA among groups, and by *t*-test between two groups. *P* < 0.05 was considered statistically significant.

## RESULTS

### Notch-1^MAC-KO^ protected mice against HFD-induced NAFLD

**Figure 1:**
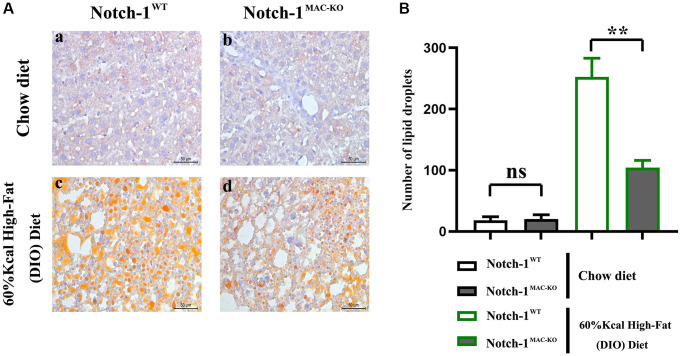
**Effect of Notch-1^MAC-KO^ on HFD-induced NAFLD mice.** (**A**) Graphs of oil red O staining results. (**B**) Lipid droplet counting statistics. (^**^P < 0.01; *N* = 6).

NAFLD is a liver disease associated with HFD. HFD contains a lot of saturated fat and cholesterol, and long-term excessive intake of these substances can lead to fat accumulation in the liver, ultimately leading to NAFLD. When fat intake exceeds the liver’s ability to process it, fat accumulation will be caused in the liver. If there are no control measures, inflammatory responses may be then triggered, leading to inflammation and damage to the liver, and contributing to the progression of NAFLD [2]. To investigate the regulatory effect of Notch-1^MAC-KO^ on lipid metabolism in HFD-induced NAFLD mice, lipid deposition in liver tissues was detected by oil red O staining. It was observed that lipid deposition in mice with NAFLD was reduced in the Notch-1^MAC-KO^ group (Figure 1), suggesting that Notch-1^MAC-KO^ may have a protective effect against HFD-induced NAFLD.

### Notch-1^MAC-KO^ regulated the expressions of related factors in macrophages of HFD-induced NAFLD mice

**Figure 2:**
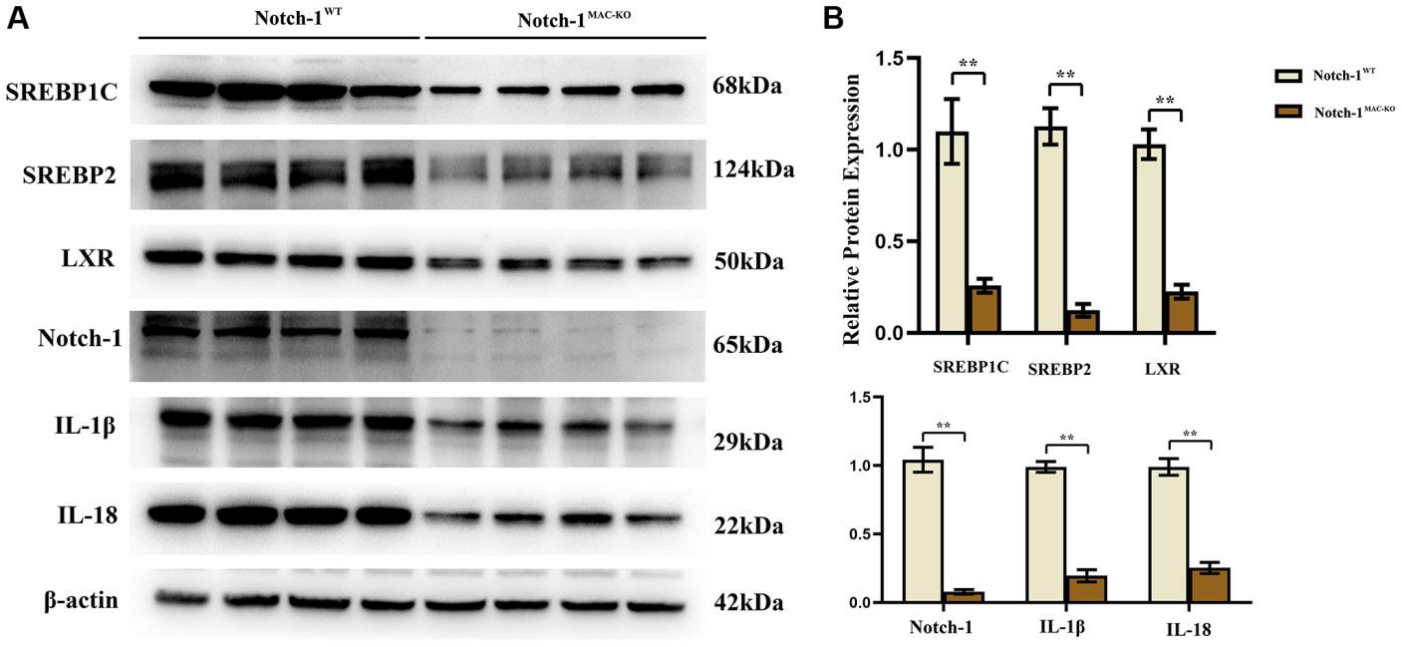
**Notch-1^MAC-KO^ regulates the expression of inflammation-related factors and lipid synthesis proteins in macrophages of HFD-induced NAFLD mice.** (**A**) Protein band plots of SREBP1C, SREBP2, LXR, IL-1β, IL-18 and Notch-1 in macrophages. (**B**) Relative protein expression of SREBP1C, SREBP2, LXR, IL-1β, IL-18 and Notch-1 in macrophages. (^**^p < 0.01, ^*^p < 0.05, ns: *p* > 0.05; *N* = 6).

**Figure 3:**
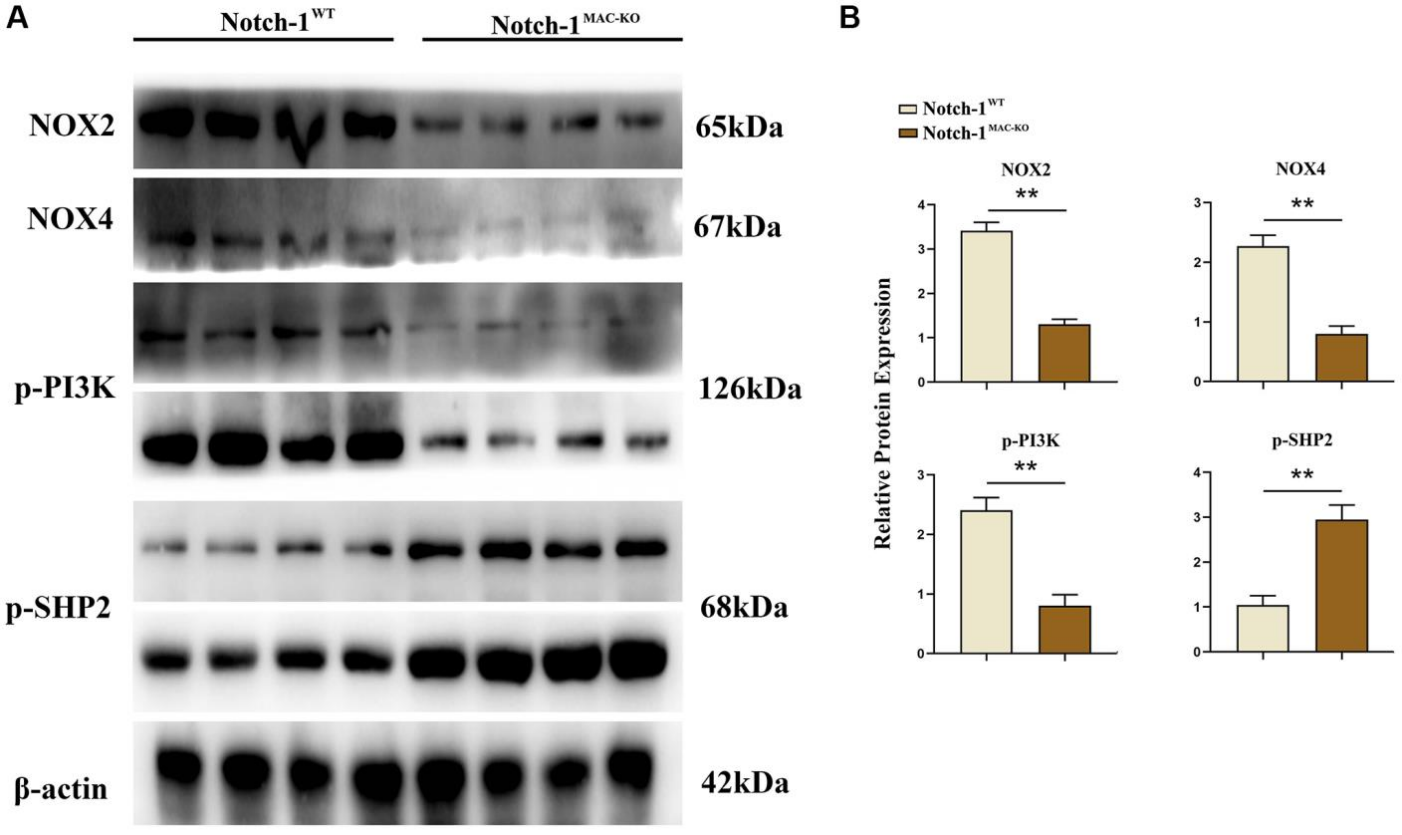
**Notch-1^MAC-KO^ regulates the expression of oxidative stress-related factors and related proteins in macrophages of HFD-induced NAFLD mice.** (**A**) Protein band plots of NOX2, NOX4, p-PI3K and p-SHP2 in macrophages. (**B**) Relative protein expression of NOX2, NOX4, p-PI3K and p-SHP2 in macrophages. (^**^p < 0.01, ^*^p < 0.05, ns: *p* > 0.05; *N* = 6).

Several molecular and inflammation-related factors are thought to be involved in the development and progression of NAFLD, including SREBP2, LXR, IL-1β, and IL-18. SREBP2 and LXR are implicated in the regulation of lipid metabolism in the liver, while IL-1β and IL-18 are implicated in the regulation of hepatic inflammatory responses. These factors have complex interactions and regulatory networks, which play an important role in the development and progression of NAFLD [5, 6]. The results of Western blotting showed that in macrophages, the protein expressions of SREBP1C, SREBP2, LXR, IL-1β, IL-18, Notch-1, NOX2, NOX4 and p-PI3K significantly decreased, while the protein expression of p-SHP2 significantly increased in the Notch-1^MAC-KO^ group compared with the Notch-1^WT^ group (Figures 2 and 3). It can be seen that specific knockout of Notch-1 can inhibit lipid synthesis and inflammatory factors by promoting SHP2 phosphorylation, thus protecting mice against NAFLD.

### Notch-1^MAC-KO^ reduced inflammation-related factors in macrophage exosomes of HFD-induced NAFLD mice

**Figure 4:**
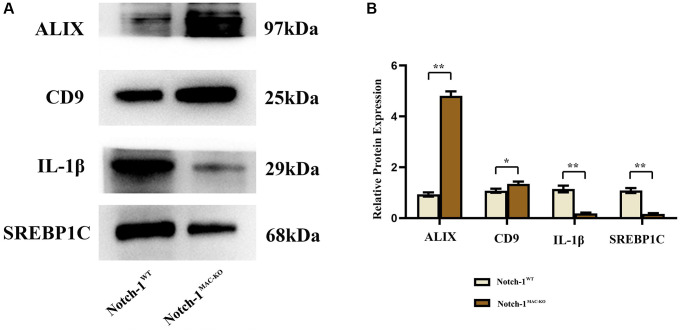
**Notch-1^MAC-KO^ reduces inflammation-related factors in exosomes of macrophages from mice with HFD-induced NAFLD.** (**A**) Protein band plots of ALIX, CD-9, IL-1β and SREBP1C in exosomes. (**B**) Relative protein expression levels of ALIX, CD-9, IL-1β and SREBP1C were detected in exosomes isolated from macrophages. (^**^p < 0.01, ^*^p < 0.05, ns: *p* > 0.05; *N* = 6).

There is growing evidence that exosomes play a crucial role in cellular communication by transporting a group of cellular components from different cells to recipient cells [10]. To determine the role of exosomes in the communication between macrophages and hepatocytes in NAFLD mice, the expressions of inflammation-related factors in macrophage exosomes were detected by Western blotting. The results revealed that the expressions of ALIX, CD9, IL-1β and SREBP1C in macrophage exosomes were significantly lower in the Notch-1^MAC-KO^ group than in the Notch-1^WT^ group (Figure 4).

### Specific knockout of Notch-1 inhibited inflammatory factors and lipid synthesis in NAFLD mice by promoting SHP2 phosphorylation

**Figure 5:**
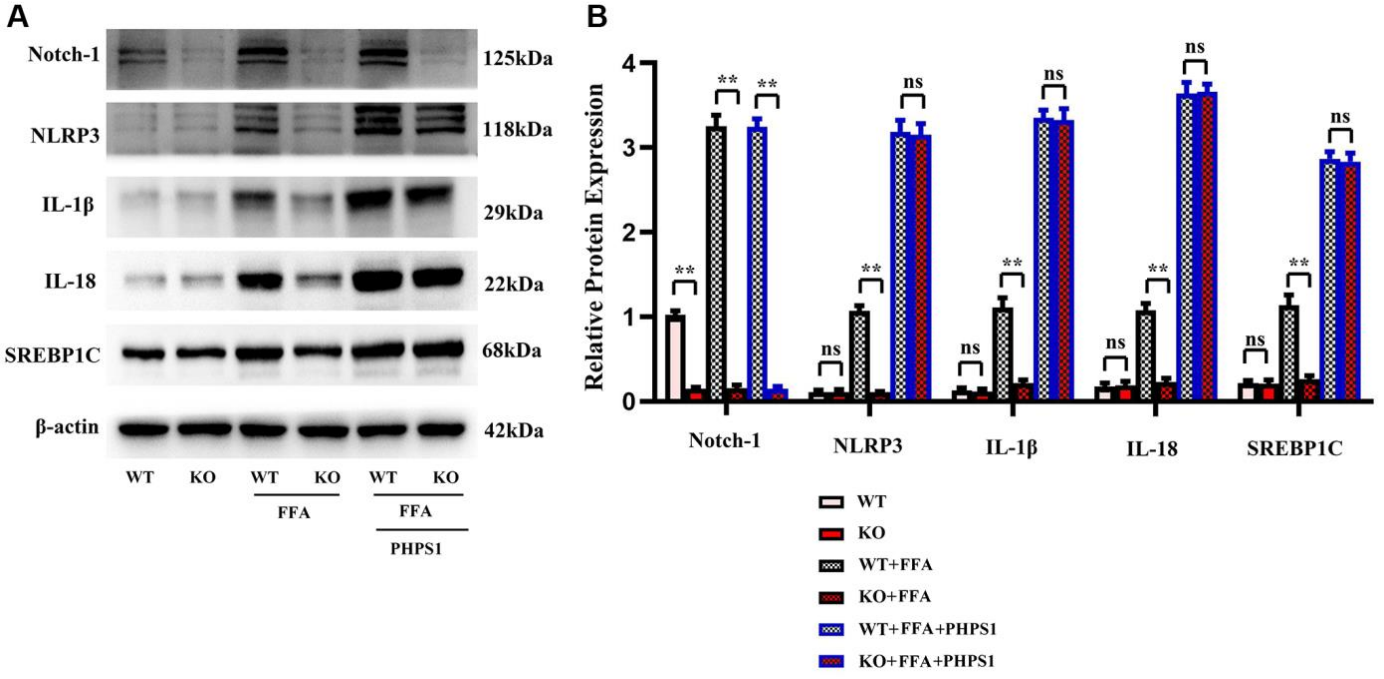
***In vitro* experiments verified that Notch-1^MAC-KO^ regulated the correlation between the expression of inflammation-related factors and lipid synthesis in macrophages of HFD-induced NAFLD mice.** (**A**) Protein band plots of Notch-1, NLRP3, IL-1β, IL-18 and SREBP1C. (**B**) Relative protein expression levels of Notch-1, NLRP3, IL-1β, IL-18 and SREBP1C. (^**^p < 0.01, ^*^p < 0.05, ns: *p* > 0.05; *N* = 3).

**Figure 6:**
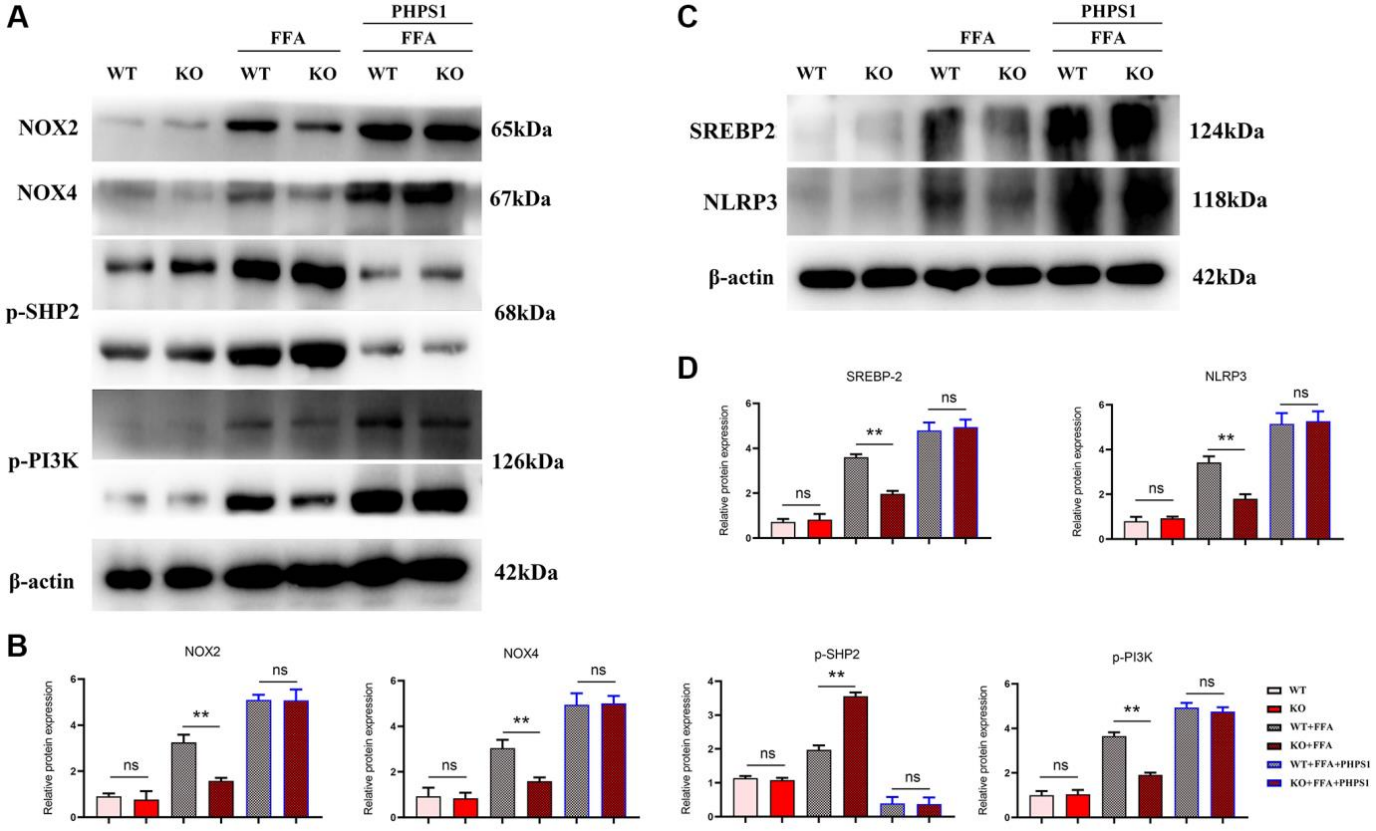
***In vitro* experiments verified that Notch-1^MAC-KO^ regulated the expression of oxidative stress-related factors and associated proteins in macrophages of HFD-induced NAFLD mice.** (**A**) Protein band plots of NOX2, NOX4, NLRP3, p-PI3K, p-SHP2 and SREBP2; (**B**) Relative protein expression levels of NOX2, NOX4, p-PI3K, NLRP3, p-SHP2 and SREBP2; (**C**) Protein band plots of NOX2, NLEP3, p-PI3K, p-SHP2 and SREBP2; (**D**) Relative protein expression levels of NOX2, NLEP3, p-PI3K, p-SHP2 and SREBP2. (^**^p < 0.01, ^*^p < 0.05, ns: *p* > 0.05; *N* = 3).

**Figure 7:**
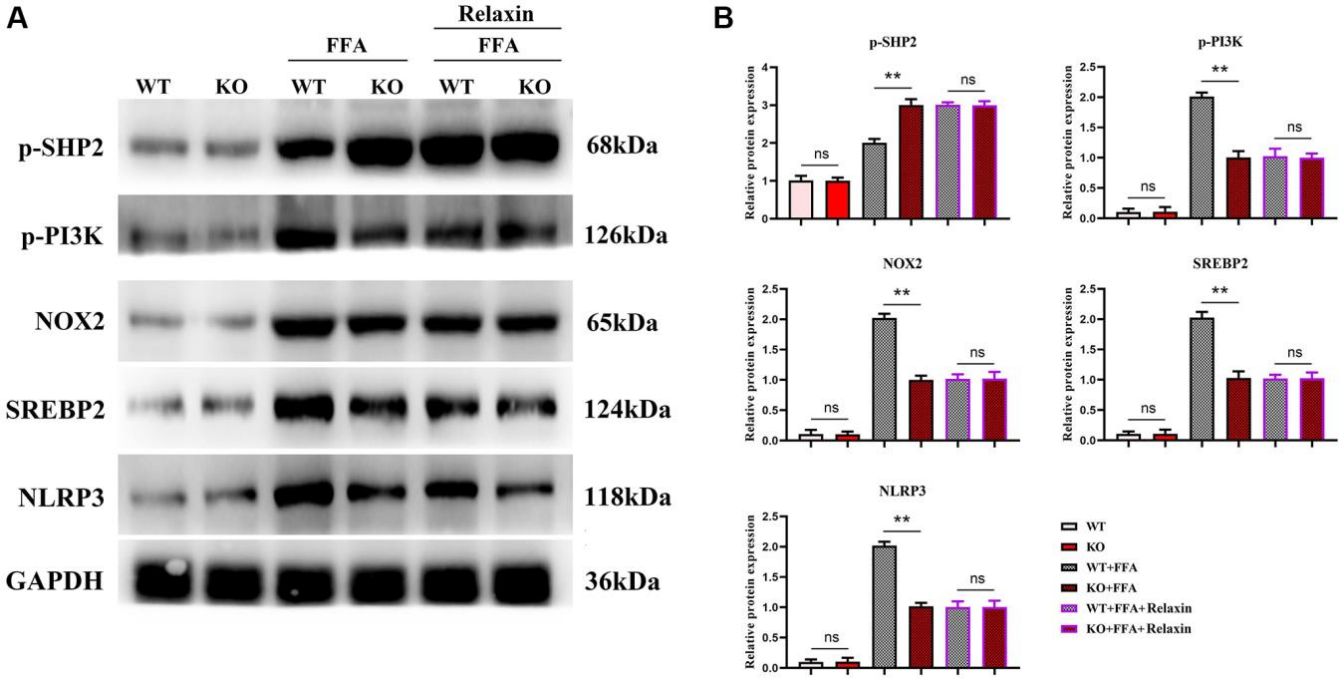
***In vitro* experiments have confirmed that Relaxin has a protective effect on the liver from anti-inflammation.** (**A**) Protein band plots of NOX2, NLEP3, p-PI3K, p-SHP2 and SREBP2; (**B**) Relative protein expression levels of NOX2, NLEP3, p-PI3K, p-SHP2 and SREBP2. (^**^p < 0.01, ^*^p < 0.05, ns: *p* > 0.05; *N* = 3).

**Figure 8:**
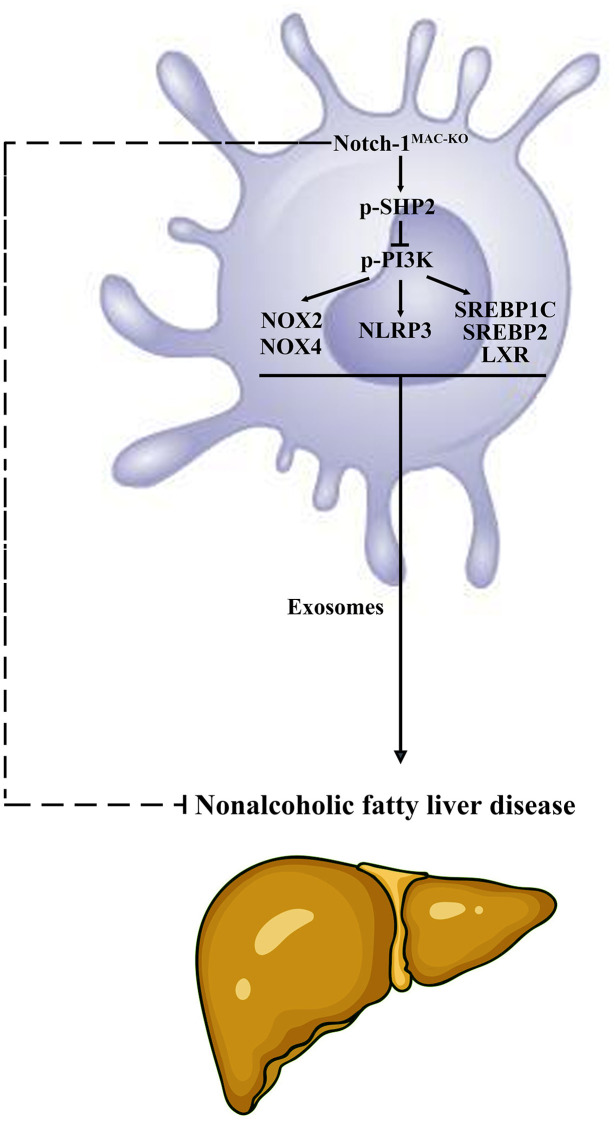
**Specific knockout of Notch-1 in mice facilitates the phosphorylation of SHP2 in macrophages to suppress inflammatory factors, lipid synthesis and exosome secretion.** This reduces the secretion of macrophage exosomes carrying inflammatory factors and lipid-synthesizing proteins to hepatocytes, thus protecting against NAFLD in mice.

Studies have shown that Relaxin exerts hepatoprotective effects in human and mouse liver IRI and OLT by increasing the expression of macrophage NICD and anti-inflammatory genes [19]. To validate the conclusions made by *in vitro* experiments, *in vitro* cell experiments were designed. As shown in Figure 5, the expressions of NLRP3, IL-1β, IL-18 and SREBP1C significantly decreased in the Notch-1^MAC-KO^+FFA group compared with the Notch-1^WT^+FFA group, which were significantly reversed by the SHP2 inhibitor PHPS1. As shown in Figures 6 and 7, the Notch-1^MAC-KO^+FFA group had significantly decreased expressions of NOX2, NOX4, p-PI3K, NLRP3 and SREBP2 and a significantly increased expression of p-SHP2 compared with the Notch-1^WT^+FFA group. However, the differences in the above proteins were all eliminated after PHPS1 and Relaxin were added. To sum up, specific knockout of Notch-1 inhibits inflammatory factors and lipid synthesis in NAFLD mice by promoting SHP2 phosphorylation, thereby attenuating NAFLD (Figure 8).

## DISCUSSION

Defined as hepatic steatosis without heavy drinking, NAFLD is one of the most common chronic liver diseases worldwide. The prevalence of NAFLD is increasing, about 20–30% in the global population and about 75–100% in the obese population [21]. NASH is the progressive form of NAFLD, which combines steatosis with inflammation and fibrosis, and is a major cause of end-stage liver disease. Unlike simple steatosis, NASH is irreversible and can eventually progress to fibrosis, cirrhosis or even HCC. Despite many studies on NAFLD, its specific mechanisms are unknown, and no effective treatment is available yet [22]. Therefore, it is necessary to find effective therapeutic targets for NAFLD.

The Notch pathway is one of the most commonly activated signaling pathways in human liver diseases. Since the Notch pathway regulates a variety of cellular activities, dysregulation of Notch is involved in many pathological processes. Moreover, the important and complex role of Notch signaling in the regulation of inflammatory stress in the liver has been verified. For example, the Notch-RBP-J pathway inhibits ROS production through the JAK2/STAT3 signaling pathway, thereby protecting hepatocytes from I/R injury. In the case of Notch-1 deficiency in the bone marrow, the RhoA/ROCK pathway is activated, thus worsening hepatocyte injury in mice.

NLRP3 inflammasome activation-induced inflammatory responses are critical in the development of NAFLD, particularly during the progression from NAFLD to NASH. Inflammasomes are multiprotein complexes that mediate the production of inflammatory cytokines and trigger a potent inflammatory cascade response, among which NLRP3 inflammasomes are the most widely studied and best-characterized ones. NLRP3 inflammasomes mediate many non-infectious factor-induced inflammations such as lipid accumulation, hyperglycemia-induced oxidative stress, and hyperlipidemia, which are involved in the etiology of a variety of diseases, including NAFLD, obesity, diabetes mellitus and cardiovascular disease [23–25]. Therefore, the regulation of NLRP3 inflammasomes is a major therapeutic means for NAFLD. Besides, SHP2 can dephosphorylate ANT1, maintain mitochondrial integrity, and prevent the overactivation of inflammasomes and the resulting inflammation.

In this study, Notch-1 was specifically knocked out in mice. It was found by oil red O staining that specific knockout of Notch-1 could reduce lipid deposition in the liver. The results of Western blotting showed that the expressions of ALIX, CD9, IL-1β and SREBP1C in macrophage exosomes were significantly lower in the Notch-1^MAC-KO^ group than in the Notch-1^WT^ group. In macrophages, the expressions of SREBP1C, SREBP2, LXR, IL-1β, IL-18, Notch-1, NOX2, NOX4 and p-PI3K significantly decreased, while the expression of p-SHP2 significantly increased in the Notch-1^MAC-KO^ group compared with the Notch-1^WT^ group. In *in vitro* experiments, the Notch-1^MAC-KO^+FFA group had significantly decreased expressions of SREBP1C, NLRP3, IL-1β, IL-18, SREBP2, NOX2, NOX4 and p-PI3K and a significantly increased expression of p-SHP2 compared with the Notch-1^WT^+FFA group. However, the differences in the above proteins were all eliminated after PHPS1 and Relaxin were added. These findings suggest that specific knockout of Notch-1 reduces inflammation and lipid deposition in NAFLD by promoting SHP2 phosphorylation.

In conclusion, specific knockout of Notch-1 can suppress inflammatory factors, lipid synthesis and exosome secretion by promoting SHP2 phosphorylation, thereby attenuating NAFLD.
